# Creep behavior and long-term strength characteristics of pre-peak damaged sandstone under conventional triaxial compression

**DOI:** 10.1038/s41598-023-31028-6

**Published:** 2023-03-08

**Authors:** Rongbin Hou, Qingzhe Cui, Hanhan Wu, Yanke Shi

**Affiliations:** 1 School of Civil Engineering and Communication, North China University of Water Resources and Electric Power, Zhengzhou, 450045 Henan China; 2 Guangxi Key Laboratory of Disaster Prevention and Engineering Safety, Guangxi University, Nanning, 530004 Guangxi China

**Keywords:** Solid Earth sciences, Civil engineering, Energy infrastructure, Energy storage

## Abstract

A series of creep tests were carried out on sandstone specimens with different pre-peak instantaneous damage characteristics under different confining pressures. The results revealed that the creep stress was the key factor affecting the occurrence of the three stages of creep, and the steady-state creep rate increased exponentially with increasing creep stress. Under the same confining pressure, the larger the instantaneous damage of the rock specimen was, the more quickly creep failure occurred and the lower the creep failure stress was. For the pre-peak damaged rock specimens, the strain threshold for accelerating creep was the same for a given confining pressure. The strain threshold increased with increasing confining pressure. In addition, the long-term strength was determined using the isochronous stress–strain curve and the variation in the creep contribution factor. The results revealed that the long-term strength decreased gradually with increasing pre-peak instantaneous damage under lower confining pressures. However, the instantaneous damage had little effect on the long-term strength under higher confining pressures. Finally, the macro–micro-failure modes of the sandstone were analyzed according to the fracture morphology observed via scanning electron microscopy. It was found that the macroscale creep failure patterns of the sandstone specimens could be divided into a shear-dominated failure mode under high confining pressures and a mixed shear-tensile failure mode under low confining pressures. At the microscale, as the confining pressure increased, the micro-fracture mode of the sandstone changed gradually from a single brittle fracture to a mixed brittle and ductile fracture mode.

## Introduction

The term “creep” describes the phenomenon that the deformation of solid materials increases with time under constant stress. In underground engineering, the creep properties and long-term strength of rocks have been received a great deal of attention because they play a key role in analyzing the long-term stability of underground rock structures, such as tunnels, slopes, caverns for storing liquid natural gas, underground power plants, and radioactive waste repositories^1–6^. Besides, the creep of coal and rock is of great concern as it may cause shrinkage and collapse of boreholes, and it can even affect the gas extraction performance^7–9^. Over the past few decades, studies of creep behavior have been widely carried out on many rock types via experimental and theoretical methods, and many valuable results have been obtained^10–16^. However, with the increasing complexity of deep rock engineering, the existing research results cannot effectively support the needs of engineering construction. As is well known, in both underground rock excavation and drilling, the internal stress of the surrounding rock is redistributed, which can induce damage or fractures in the rock. Furthermore, the long-term deformation and strength characteristics of a rock mass depend on the development of the internal damage and rupture caused by complex engineering disturbances^5,13,17–19^. Many engineering applications have shown that the time-dependent deformation of the surrounding rock and the instability of the supporting structure may still occur after the completion of deep underground engineering construction^20–23^. A large number of gas drainage hole casing collapse incidents induced by creep of the surrounding rock in deep coal mining have been reported^8,24,25^. Therefore, studying the creep properties and long-term strength of rocks by considering the instantaneous damage induced by excavation unloading has theoretical and engineering significance for deep rock engineering.

Laboratory creep tests are an important research method for investigating the time-dependent behavior of rocks. Based on different research backgrounds, scholars have conducted a large number of studies to investigate the creep characteristics of rocks via laboratory creep tests^1–5,26–28^. Existing data show that the characteristics of time-dependent deformation can be divided into three stages: transient (or primary) creep, steady-state (or secondary) creep, and accelerating (or tertiary) creep^16,19^. In particular, experimental results indicate that there is a threshold stress (i.e., a long-term strength), which affects the occurrence of steady-state creep. When the applied load is greater than the threshold stress, the steady-state creep with a uniform strain rate is positively correlated with the stress level^29–31^. In addition, scholars have pointed out that the confining pressure and external environment (e.g., water, temperature, and chemistry) both significantly affect creep behavior. Zhang et al.^8^ conducted creep tests on deep coal under constant axial pressure and unloading confining pressure at different temperatures and analyzed the characteristics of the resultant creep deformation. Their experimental results revealed that the coal samples with higher temperatures experienced greater axial deformation; however, the radial deformation did not change monotonically with the change in temperature. To study the effect of coupled axial and hydraulic pressure on the creep behavior of sandstone, Liu et al.^11^ used a multi-channel fluid–solid coupling rock rheology system to carry out multi-loading creep experiments and reported that the creep strain and the acceleration creep rate were affected by the hydraulic pressure under both the same initial load and multi-step loading. Yu et al.^28^ found that soaking rock specimens led to significant increases in the creep strain and the creep strain rate compared with those of dry specimens, and the long-term strength was much lower due to the effect of immersion. In recent years, some scholars have pointed out that determining the effect of dynamic disturbances on creep is a vital prerequisite for understanding the time-lag properties of rockbursts^18,27,32^. Several valuable experimental results have been obtained and detailed theoretical models have been proposed. Zhu et al.^18^ performed creep experiments on sandstone under a dynamic disturbance using a new creep-impact testing machine. Their results showed that a dynamic disturbance introduces further damage to the rock and decreases the time it takes for creep failure to occur, resulting in a higher axial strain rate, absolute volumetric strain rate, and acoustic emission (AE) energy rate.

The above outstanding achievements have notably increased our understanding of the time-dependent behavior of rocks under various external factors. However, previous research on the creep characteristics and long-term strength of rocks mainly focused on intact rock samples or post-peak rupture rock samples, and little research has been conducted on pre-peak damaged rock specimens. Additionally, in underground engineering, the surrounding rock is usually in a three-dimensional stress state. Therefore, studying the influence of the pre-peak instantaneous damage of the rock on the creep properties and long-term strength under triaxial conditions has guiding significance for determining the time-dependent damage mechanism of deep surrounding rock. In this study, sandstone specimens with different degrees of damage were prepared via loading and unloading tests. Then, multistep creep tests under different confining pressures were conducted, and the effects of the instantaneous damage degree and stress conditions on the strain, creep rate, time to failure, and critical state of creep failure were investigated. Next, the long-term strengths of the various damaged sandstone specimens were determined, and the effects of the confining pressure and initial damage degree were analyzed. Finally, the macro- and micro-failure modes of the specimens under different confining pressures were analyzed according to the fracture morphology observed via scanning electron microscopy (SEM).

## Experimental materials and scheme

### Rock specimens and testing equipment

**Figure 1:**
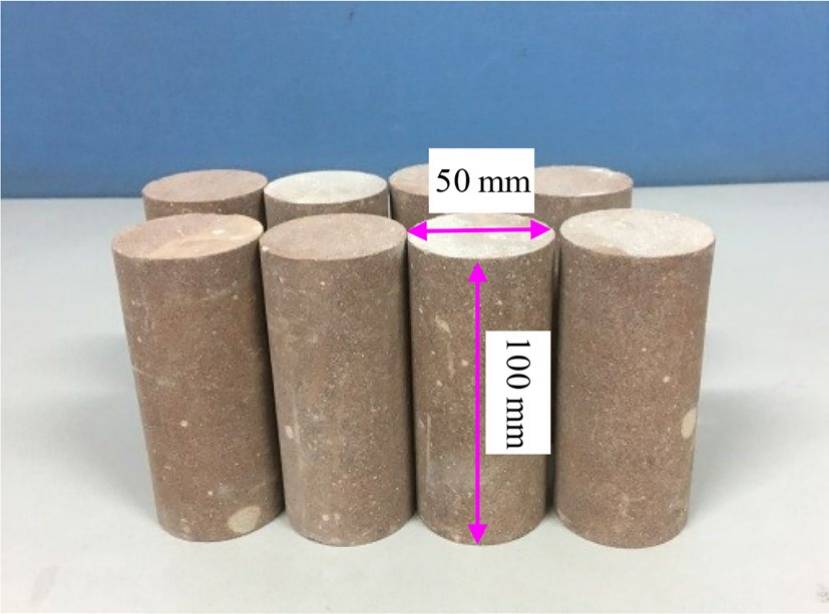
Part of sandstone specimens.

The sandstone used for the experiments was collected from Shandong Province, eastern China. This sandstone was brown–red and had a relatively dense texture. Cylindrical specimens with a diameter of 50 mm and a height of 100 mm (Fig. 1) were cored from an intact sandstone block. During the preparation of the specimens, an infrared ray was used to guide the cutting, and a surface grinder was used to polish the ends of the samples following the suggestions of the International Society for Rock Mechanics (ISRM)^33^.

Existing research has shown that the P-wave velocity is closely related to the strength, and it is an important parameter for analyzing the discreteness of rock specimens^19,34^. Thus, the P-wave velocity was measured along the axial direction of the specimens, and its average value was 2830 m/s. Then, the specimens with P-wave velocities of 2730–2930 m/s were prepared for use in the subsequent tests. In order to fully understand the basic physical and mechanical properties of the sandstone specimens, physical tests and uniaxial and triaxial compression tests were conducted following the rock testing methods suggested by the ISRM. The average values of the natural density, elastic modulus, and Poisson’s ratio were 2380 kg/m^3^, 9.68 GPa, and 0.27, respectively. The uniaxial and triaxial compression tests (confining pressures of 5, 10, and 20 MPa) were carried out at a loading rate of 0.05 MPa/s. The test results provided the important basic parameters for the subsequent experimental design of the damaged sandstone samples (Table 1).

**Table 1:** Compressive strength of specimens under different confining pressures.

Number of specimens	Confining pressure (MPa)	Average of peak strength (MPa)
3	0	39.2
3	5	63.2
3	10	78.8
3	20	117.4

**Figure 2:**
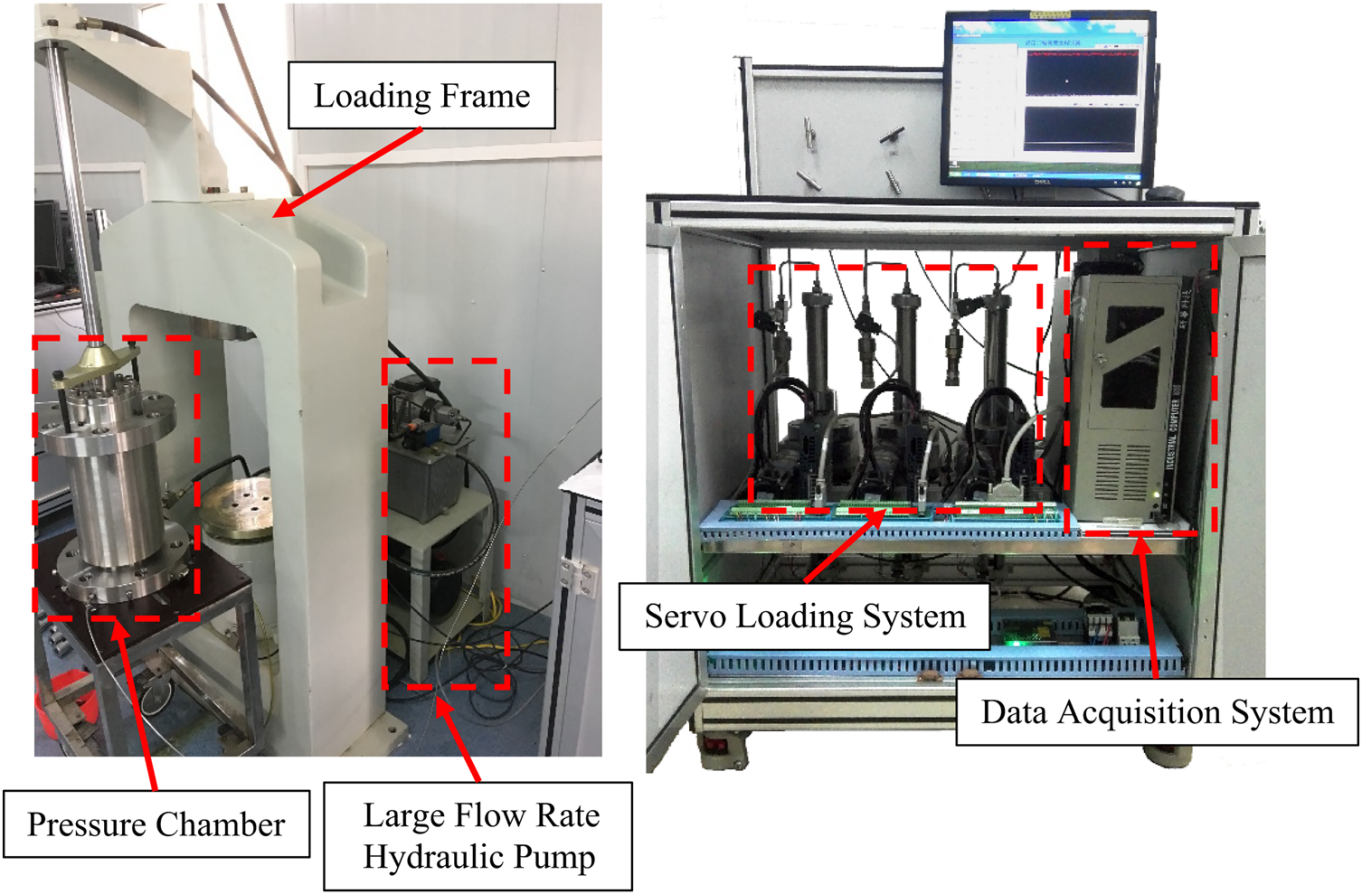
THMC multi-field coupling test equipment of rock.

In this study, all of the mechanical tests were conducted using a servo thermal-hydro-mechanical-chemical (THMC) multi-field coupling rock testing instrument designed by the Institute of Rock and Soil Mechanics, Chinese Academy of Sciences (Fig. 2). The experimental equipment consisted of three key components: a pressure chamber, servo loading system, and data acquisition system. The axial loading force of the experimental equipment reached 1500 kN, and it was maintained at a constant value by the servo loading system. The designed pressure standard of the pressure chamber was 100 MPa. During the tests, the deformation of the specimen, the axial load, and the confining pressure were automatically recorded by the data acquisition system.

### Experimental scheme

To investigate the creep characteristics and long-term strength of damaged rock after engineering disturbance, the preparation of the damaged samples was the primary work in this study. It is difficult to evaluate the degree of damage of rock specimens obtained directly from the surrounding rock because of the complex geological environment and engineering activities on-site. Previous studies have shown that the loading–unloading test is an effective method of prefabricating damage in rock specimens^13,17^. In addition, most of the surrounding rock will be in a pre-peak damaged state due to the release of the in-situ stress after rock mass excavation. Therefore, selecting different pre-peak unloading points based on the characteristics of the rock stress–strain curve is one of the key steps in loading–unloading tests for producing damaged specimens.

The crack initiation stress is usually 40–60% of the compressive strength^35^. Thus, the stresses of the unloading points were set to more than 60% of the compressive strength under different confining pressures, including 65%, 80%, 90%, and 97% (Table 2). Moreover, in order to reduce the influence of the differences between the specimens on the test results, five specimens without fissures, breakage, or impurities and with approximately the same P-wave velocity were selected to conduct the loading–unloading tests under each of the testing scenarios. As well known, natural rocks are heterogeneous bodies, which are filled with various initial defects such as micro-cracks. Moreover, the initial defects of the rock specimens are also influenced by the sampling process greatly. Thus, in order to accurately evaluate the instantaneous damage of specimen induced by the external load, first, the compressive deformation of initial defects of rock specimen should be obtained via loading–unloading test. By this way, the axial stress of the specimen is applied to 30% of the peak strength, and then unloaded completely to obtain the unrecoverable strain caused by crack compaction. After that, according to the unloading stress in Table 2, a second loading–unloading test was conducted out to prepare the damaged specimens. In the case, the specimens were loaded to the predetermined stress level and were completely unloaded after 5 min to obtain the plastic deformation caused by the material yield flow. Finally, the three damaged specimens with the closest P-wave velocities (obtained via acoustic testing) were selected for the subsequent creep tests.

**Table 2:** Unloading stress and creep stress levels under different confining pressure.

Damaged specimens no	Confining pressure (MPa)	Unloading stress (MPa)	Creep stress (MPa)						
			1st	2nd	3rd	4th	5th	6th	7th
5D_1_	5	41.1	25.3	34.8	44.2	50.6	53.7	56.9	60.1
5D_2_	5	50.6	25.3	34.8	44.2	50.6	53.7	56.9	60.1
5D_3_	5	56.9	25.3	34.8	44.2	50.6	53.7	56.9	60.1
5D_4_	5	61.9	25.3	34.8	44.2	50.6	53.7	56.9	60.1
10D_1_	10	51.2	31.5	43.3	55.2	63.1	67.0	70.9	74.9
10D_2_	10	63.1	31.5	43.3	55.2	63.1	67.0	70.9	74.9
10D_3_	10	70.9	31.5	43.3	55.2	63.1	67.0	70.9	74.9
10D_4_	10	77.2	31.5	43.3	55.2	63.1	67.0	70.9	74.9
20D_1_	20	76.3	47.0	64.6	82.2	93.9	99.8	105.7	111.5
20D_2_	20	93.9	47.0	64.6	82.2	93.9	99.8	105.7	111.5
20D_3_	20	105.7	47.0	64.6	82.2	93.9	99.8	105.7	111.5
20D_4_	20	115.1	47.0	64.6	82.2	93.9	99.8	105.7	111.5

**Figure 3:**
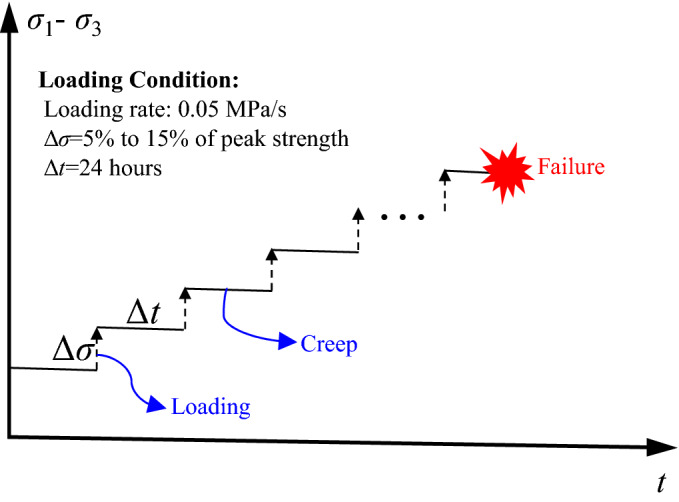
Schematic diagram of the triaxial creep test.

The triaxial creep tests conducted in this study were carried out using the multistage loading method and were performed at room temperature (25 ± 1 °C). Seven creep stress levels were set based on the compressive strength of the rock specimens (Table 2). A schematic diagram of the triaxial creep test is presented in Fig. 3. Additionally, a preliminary creep test was conducted on the intact rock specimens before the creep tests on the damaged specimens. The results showed that the duration of each stress level should be constant for more than 24 h. The experimental process was as follows.

- First, we wrapped the specimens in a thin rubber film to prevent the oil from immersing them. Both ends of the specimens were coated with petrolatum, and the specimen was placed in the pressure chamber.
- Then, the pressure chamber was filled with oil via a large flow rate hydraulic pump, and the servo pump was used to apply the target confining pressure at a rate of 0.05 MPa/s.
- Finally, the axial stress was applied to the specified creep stress levels at a loading rate of 0.05 MPa/s using a multi-stage procedure until the specimen failed (Fig. 3).

### Definition of initial instantaneous damage of specimens

The damage variable is an index used to describe the internal damage state of a rock, and it can be used to quantitatively analyze the occurrence and growth of cracks during the process of rock failure. It is considered to be a key indicator for rock failure warning^34–36^. It is very important to define the damage variable through the variation in the macroscopic mechanical parameters (such as the deformation, AE events, and elastic modulus)^37–42^. Based on the variations in the strain and elastic modulus, the damage evolution equation proposed by Xie et al. has been widely used^42^:

1 $$D = 1 - \frac{{\varepsilon - \varepsilon^{\prime}}}{\varepsilon }\left( {\frac{{E^{\prime}}}{E}} \right)$$

where *D* is the damage variable, *ε* is the total strain, *ε*′ is the residual strain after unloading, *E* is the elastic modulus, and *E*′ is the unloading modulus.

In Eq. (1), the residual strain consists of two parts, one is the irrecoverable strain caused by crack compaction, and the other is the plastic strain caused by the material yield flow. The crack compaction deformation is essentially related to the geometric properties and the crack density. As the primary state of each specimen is different, the crack compaction deformation of the rock specimen may affect the results for evaluating the degree of the instantaneous damage. Therefore, in this paper, the damage variable equation was established based on Eq. (1) by ignoring the crack compaction strain:

2 $$D_{{\text{c}}} = 1 - \frac{{\varepsilon - \varepsilon_{r} }}{{\varepsilon - \varepsilon_{u} }}\left( {\frac{{E^{\prime}}}{E}} \right)$$

where *D*_c_ is the instantaneous damage caused by the stress variation, *ε*_u_ is the crack compression strain obtained by the first loading–unloading test, and *ε*_r_ is the total residual strain contained the crack compression strain and the plastic strain caused by the material yield.

**Figure 4:**
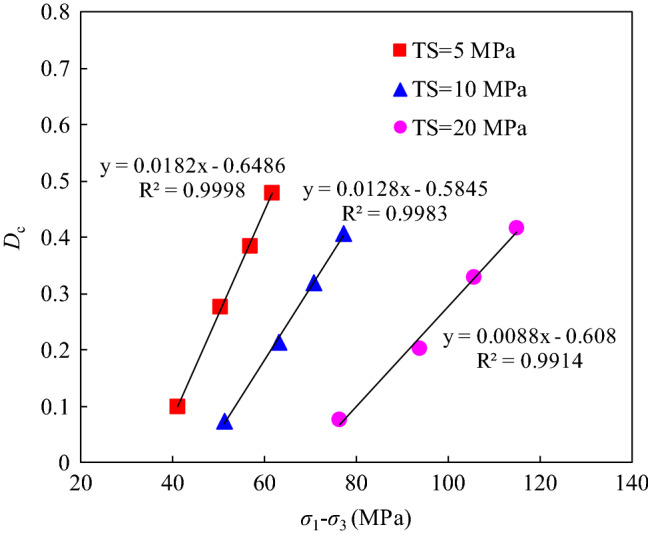
Relationship between the axial unloading stress and instantaneous damage under different confining pressure.

The instantaneous damage coefficients of each specimen were calculated based on the results of the loading–unloading tests. The relationship between the unloading stress and the instantaneous damage under different confining pressures is shown in Fig. 4. The results show that the instantaneous damage increased linearly with increasing unloading stress.

## Creep experiment results and discussion

### Time-dependent deformation

**Figure 5:**
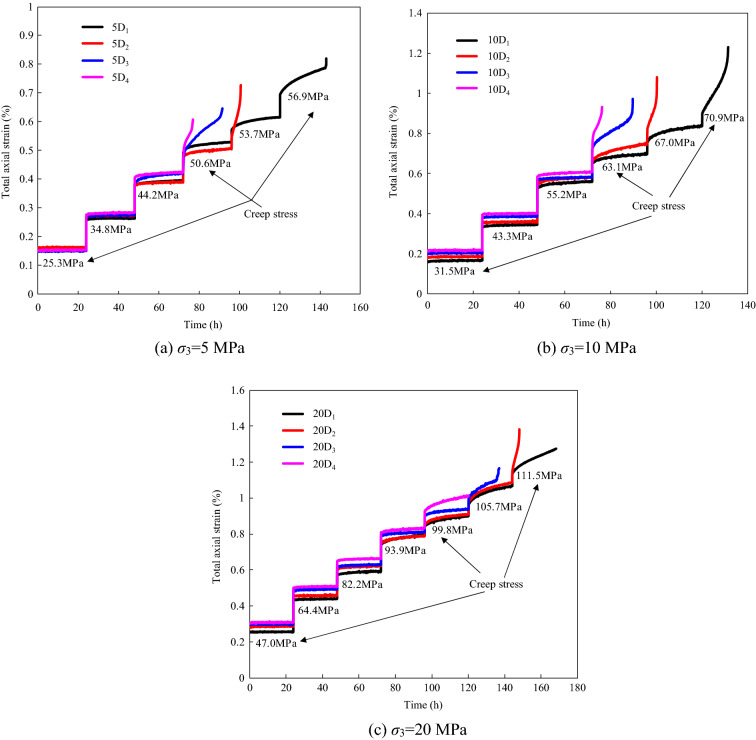
Axial strain curves of sandstone with different initial damage under multi-stage axial creep stress.

**Figure 6:**
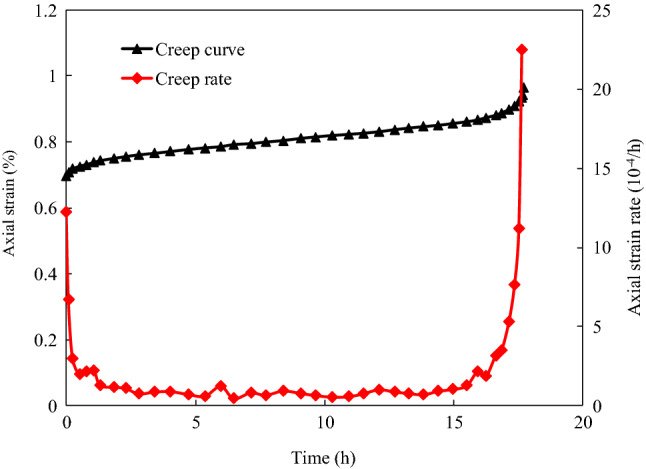
Variation of axial strain and creep rate with time of damaged specimens (10D_3_) before failure.

The strain–time curves of sandstone specimens with different degrees of instantaneous damage under confining pressures of 5, 10, and 20 MPa are shown in Fig. 5. It can be seen from Fig. 5 that the total strain consists of the instantaneous strain and the creep strain. The instantaneous strain is related to the creep stress level, and it increases with increasing stress level. Additionally, the time-dependent deformation of the sandstone specimens exhibits three distinct creep phases before failure. Figure 6 shows the evolution of axial deformation and creep rate with time at the last creep stress level before creep failure of specimen 10D_3_. As shown in Fig. 6, (1) the transient creep stage, which is characterized by an initially rapid strain rate that slows with time; (2) the steady-state creep stage, which is characterized by a constant strain rate; and (3) the accelerating creep stage, which is characterized by a nonlinearly increasing strain rate until the rock specimen fails. The test results also show that the occurrence of the different creep phases is affected by the creep stress level. At a low creep stress level, only the transient creep stage, in which the deformation gradually becomes stable with time, appears after the instantaneous deformation. As the creep stress increases, the steady-state creep stage occurs after the transient creep stage. Moreover, obvious creep deformation appears for the last creep level, which induces the accelerating creep stage.

The test results also show that the time-dependent deformations of the specimens with different degrees of instantaneous damage are significantly different under the same confining pressure. When the creep stress level is lower than the creep failure stress, the creep characteristics of the different damaged specimens exhibit approximately the same response at the same creep stress level. However, when the creep stress exceeds the creep failure stress threshold, the time-dependent deformation characteristics of the sandstone specimens are affected by both the instantaneous damage and the time-dependent damage. Taking the creep test results for the different damaged sandstone specimens under a confining pressure of 5 MPa as an example, the specimens (5D_1_ and 5D_2_) with less instantaneous damage exhibited transient creep and steady-state creep at the creep stress level of 50.6 MPa, while the specimens (5D_3_ and 5D_4_) with greater instantaneous damage exhibited all three stages of creep for the same stress level. In general, the creep behavior and damage propagation of the damaged specimens are affect by the stress level. When the stress level is lower than the long-term strength of rock, the time-dependent deformation exhibits the characteristics of stable creep, that is, the strain remains constant with time after the transient creep stage. At this time, the damage of rock will not accumulate with time and induce failure. When the loading stress level exceeds the long-term strength of rock, the rock exhibits the characteristics of unstable creep, the deformation increases over time until instability failure occurs (Fig. 6). In this process, the accumulation of damage in the rock is the main factor causing accelerating creep and failure.

**Figure 7:**
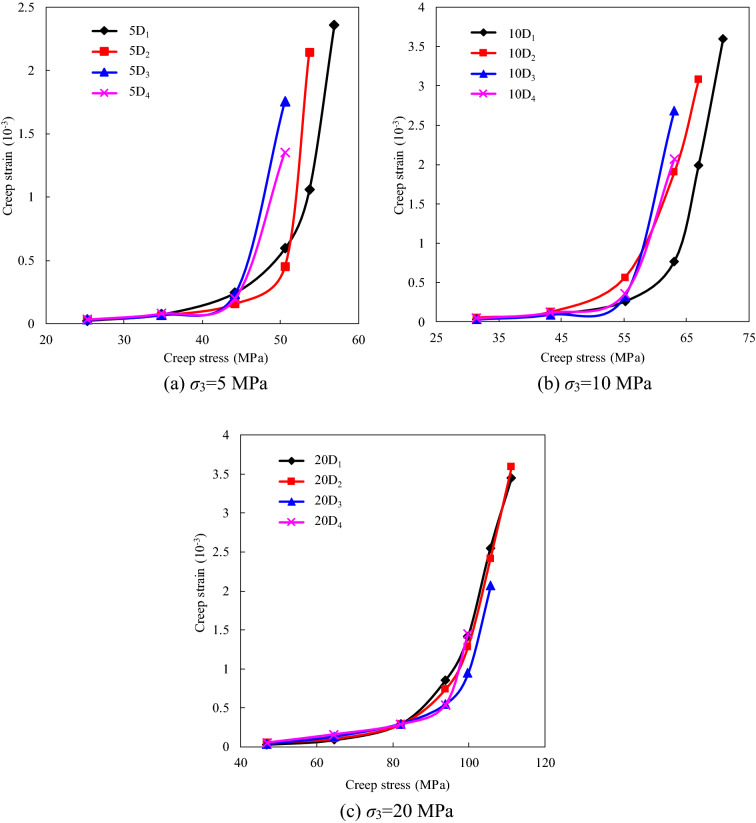
Relationship between creep strain and creep stress under different confining pressure.

**Figure 8:**
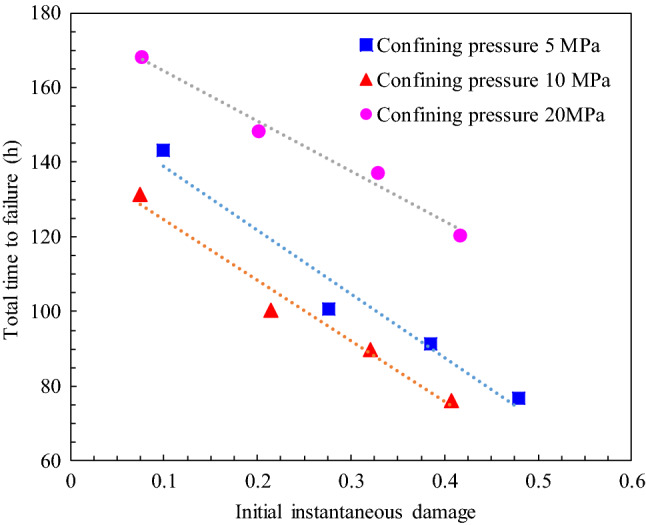
Total time to failure of the specimens with different initial instantaneous damage.

Based on the general Boltzmann superposition theorem, the creep test data were processed to obtain the one-step loading creep strain at each creep stress level (Table 3). The relationship between the creep strain and the creep stress of the specimens with different degrees of instantaneous damage is shown in Fig. 8, which shows that the creep strain increases nonlinearly with increasing creep stress. Taking the results for specimen 5D_1_ as an example, for creep stress values of 25.3, 44.2, and 50.6 MPa, the creep strains are 0.023 × 10^−3^, 0.242 × 10^−3^, and 0.594 × 10^−3^, and this lasts for 24 h. It can be seen that the creep strain increases by about 10 times when the creep stress increases from 25.3 to 44.2 MPa. As a result, the stress level is a factor that must be paid attention to in the analysis of the time-dependent deformation of rocks. Moreover, it can be seen from Fig. 7a,b that the time-dependent deformation at a higher creep stress level can be promoted by the initial degree of instantaneous damage, but this effect is weaker under higher confining pressures (Fig. 8c).

**Table 3:** Creep strain in each creep stress level under different confining pressure.

Damaged specimens no	Confining pressure (MPa)	Creep strain (10^−3^)						
		40%*σ*_c_	55%*σ*_c_	70%*σ*_c_	80%*σ*_c_	85%*σ*_c_	90%*σ*_c_	95%*σ*_c_
5D_1_	5	0.023	0.072	0.242	0.594	1.054	2.354	
5D_2_	5	0.027	0.063	0.154	0.447	2.138		
5D_3_	5	0.030	0.068	0.231	1.752			
5D_4_	5	0.034	0.079	0.196	1.351			
10D_1_	10	0.029	0.091	0.257	0.764	1.992	3.593	
10D_2_	10	0.053	0.125	0.563	1.901	3.078		
10D_3_	10	0.032	0.093	0.382	2.689			
10D_4_	10	0.048	0.117	0.360	2.071			
20D_1_	20	0.032	0.090	0.296	0.855	1.412	2.548	3.448
20D_2_	20	0.059	0.104	0.292	0.741	1.283	2.413	3.598
20D_3_	20	0.034	0.129	0.297	0.546	0.947	2.073	
20D_4_	20	0.051	0.157	0.286	0.541	1.448

### Time to failure during creep tests

As shown in Fig. 5, most of the sandstone specimens experienced three distinct creep stages and the creep was terminated by a rupture in the accelerating creep stage, except for specimens 5D_1_, 20D_1_, and 20D_4_, which were terminated in the loading section. The time to failures and the failure types of the different specimens under various confining pressure are summarized in Table 4. In addition, Fig. 8 illustrates the relationship between the total time to failure and the coefficient of the initial instantaneous damage of the sandstone specimens under different confining pressures. The results show that the time to failure of the specimens decreases significantly with increasing initial instantaneous damage under the same confining pressure. For example, when the confining pressure was 10 MPa, the specimen with the maximum instantaneous damage had the shortest time to failure (76.37 h), which was much shorter than the time to failure of the specimen with the minimum damage. The shortest time to failure of the specimens was only 58% of the longest time to failure, indicating that the initial instantaneous damage evidently affects the time to failure of the rock. Apparently, in rock engineering, it is dangerous to not consider the influence of the instantaneous damage on the time-dependent behavior of the rock because the long-term stability of the rock mass will be overestimated.

**Table 4:** Time to failure of damaged specimens under different confining pressure.

Damaged specimens no	Confining pressure (MPa)	Total time to failure (h)	Time to failure at last stress level (h)	Failure type
5D_1_	5	143.10	0	In the loading section
5D_2_		100.63	4.46	Accelerating creep
5D_3_		91.57	18.64	Accelerating creep
5D_4_		76.92	4.75	Accelerating creep
10D_1_	10	131.51	11.28	Accelerating creep
10D_2_		100.37	4.14	Accelerating creep
10D_3_		89.80	17.67	Accelerating creep
10D_4_		76.37	4.21	Accelerating creep
20D_1_	20	168.21	0	In the loading section
20D_2_		148.06	3.83	Accelerating creep
20D_3_		136.96	16.79	Accelerating creep
20D_4_		120.15	0	In the loading section

### Steady-state creep rate

The creep rate is an important index for studying the creep characteristics of materials. Three stages of creep (i.e., transient creep, steady-state creep, and accelerating creep) can be distinguished by analyzing the various features of the creep rate. As a result, it can be seen from Fig. 5 that the duration of the steady-state creep stage is much longer than those of the transient creep and accelerating creep stages. Furthermore, the steady creep rate not only affects the magnitude of the time-deformation of the rock but also determines the occurrence time of the accelerating creep stage. Therefore, it is particularly important to identify the steady-state creep rate when evaluating the long-term stability in rock engineering.

**Figure 9:**
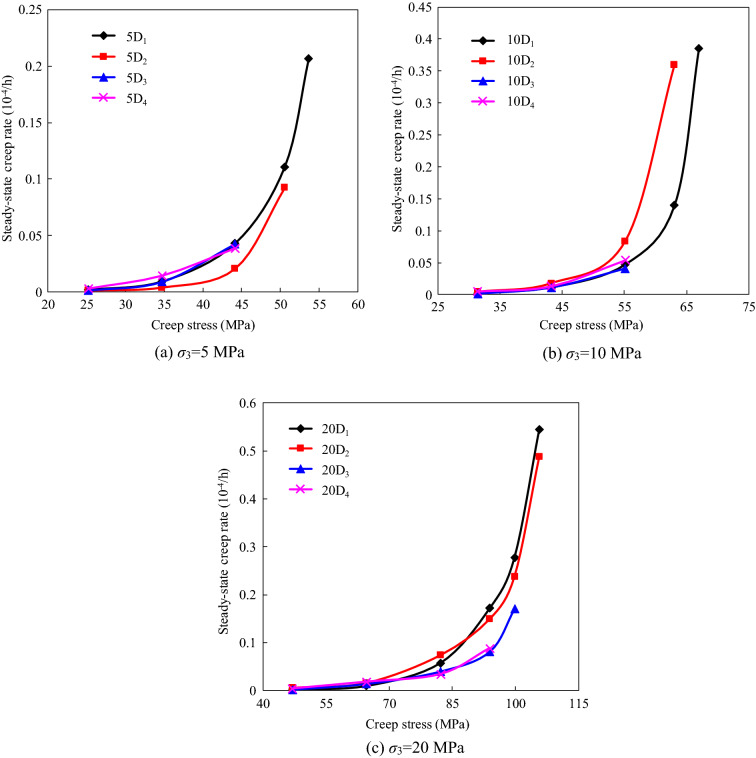
Steady state creep rate of the specimens with different initial instantaneous damage.

The steady-state creep rate can be obtained by fitting the strain–time data for the steady-state creep stage using a linear model. Figure 9 shows the relationships between the steady-state creep rate and the creep stress of the specimens with different degrees of instantaneous damage under confining pressures of 5, 10, and 20 MPa. It can be seen from Fig. 9 that the steady state creep rate increases gradually and nonlinearly with increasing creep stress level. Moreover, it can be seen that the steady-state creep rate is significantly affected by the instantaneous damage at high creep stress levels under the same confining pressure. However, since the creep stress levels under different confining pressures were different, the relationship between the steady-state rate and the confining pressure of the damaged specimens was not analyzed in this study.

### Critical state of creep failure

**Figure 10:**
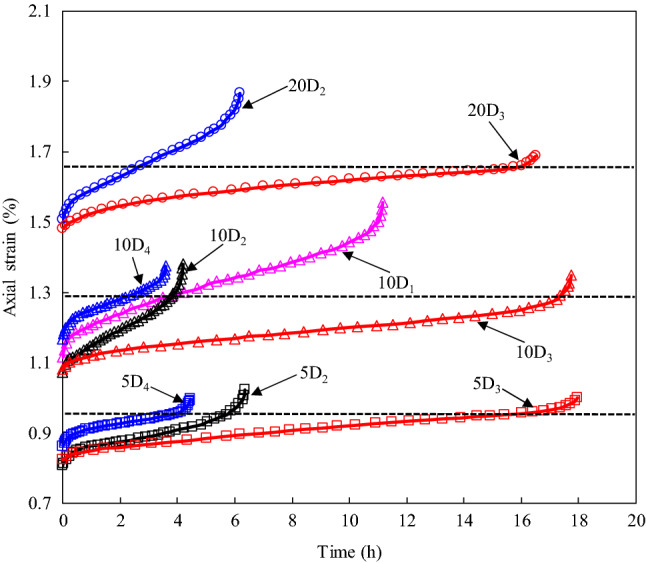
Strain–time curves of various damaged specimens at different creep failure stress level and confining pressure.

The creep experiment results verify that when the accumulation of the deformation in the transient creep and steady-state creep stages reaches a certain upper limit, the time-dependent deformation characteristics of the sandstone exhibit accelerating creep (Fig. 5). As a result, the crack propagation in the rock specimens becomes unstable, and creep failure will occur soon. Hence, researchers have regarded the attainment of the accelerating creep stage as a critical state in creep failure^6,42^. The onset of the critical state of accelerating creep has been reported based on various aspects, such as the critical damage level, critical failure stress, and critical failure strain^6,13,28,42^. In this paper, it is deemed more appropriate to discuss the critical failure state of the rock in terms of the strain threshold because of the limited number of stress levels applied in the creep tests. In order to explore the evolution of the strain with time with the creep failure stress level, the strain–time curves for the creep failure stress levels under different confining pressures were obtained (Fig. 10). It should be noted that the strain plotted in Fig. 10 includes the instantaneous strain and the creep strain under the creep load and the residual strain after the loading–unloading tests.

As Fig. 10 shows, as the confining pressure increases, a substantial increase in the axial strain occurs when the specimen fails. However, there is no significant difference in the total strain of different damaged specimens before accelerating creep under the same confining pressure condition. After that, the strain threshold corresponding to the accelerating creep of each rock sample was identified (Table 5). It can be found from Table 5 that there seems to be a unified strain threshold to control the occurrence of the accelerating creep for rock specimens with different degrees of instantaneous damage under a given confining pressure. In addition, this phenomenon is most obvious for the creep tests conducted under a confining pressure of 5 MPa.

**Table 5:** Critical strain of specimens with different instantaneous damage at different confining pressure.

Confining pressure (MPa)	Critical strain (%)					Standard deviation
	#D_1_	#D_2_	#D_3_	#D_4_	Average value	
5		0.946	0.950	0.953	0.950	0.003
10	1.432	1.281	1.258	1.309	1.320	0.067
20		1.750	1.653		1.702	0.049

**Figure 11:**
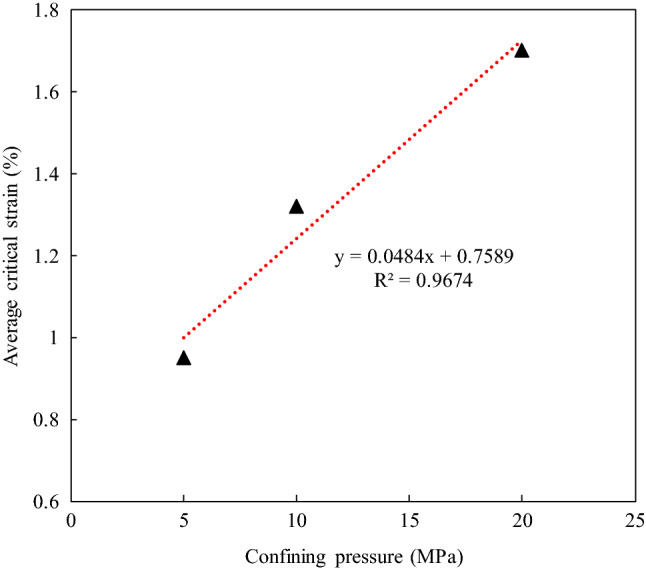
Relation between average critical strain and the confining pressure.

To further investigate the effect of the confining pressure on the critical failure state of the sandstone during creep, the average values of the critical strain at the given confining pressure were calculated. The relationship between the critical strain and the confining pressure is illustrated in Fig. 11. In Fig. 11, it can be seen that the critical strain significantly increases with increasing confining pressure, and the relationship between them can be expressed as a linear function.

## Identification of long-term strength

Previous studies have reported that a rock may fail when it is subjected to a long constant loading duration, even if the loading stress is lower than the peak strength of the rock^29^. In this case, the minimum stress that can induce the time-dependent failure is defined as the long-term strength of the rock. Engineering applications have shown that the long-term strength of a rock is a critical parameter for analyzing the long-term stability of rock engineering construction. Thus, accurately identifying the long-term strength is very important for safety analysis and lifetime prediction of rock mass structures.

### Isochronous stress–strain method

At present, many methods have been proposed for determining the long-term strength of rocks. Among them, the isochronous stress–strain curve method has been commonly used. The isochronous stress–strain curve reflects the stress–strain relationship of the rock specimens maintained under different creep stress levels for the same length of time. Based on the isochronous stress–strain curves, the stress level at the inflection point of the curves between the linear segment to the nonlinear segment is considered to be the long-term strength of the rock.

**Figure 12:**
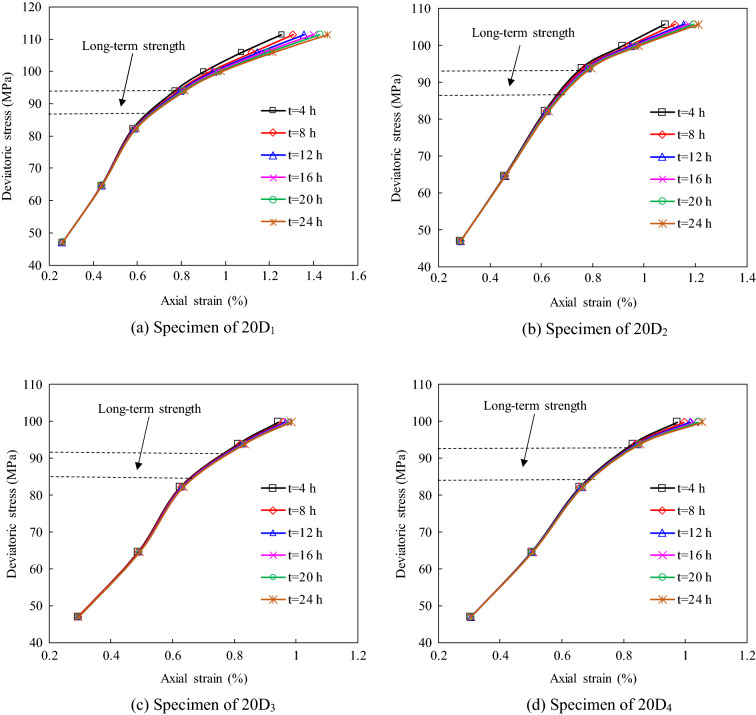
The isochronous stress–strain curves of damaged specimens at confining pressure of 20 MPa.

**Figure 13:**
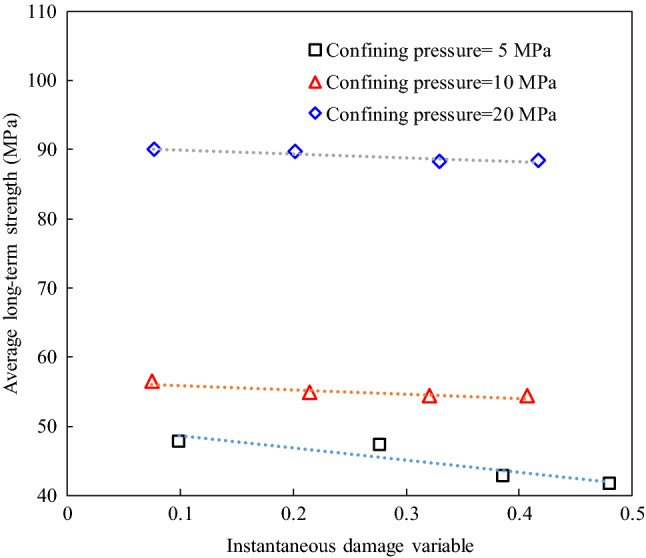
Relationship between the average long-term strength and instantaneous damage variable at different confining pressure.

In this study, the same duration (4 h) was chosen to acquire the corresponding strain data for the different creep stress levels. The isochronous stress–strain curves of the damaged specimens under confining pressures of 20 MPa were plotted using the creep stress and strain data (Fig. 12). It can be seen that the isochronous curves are a straight line at low stress levels, and then, they gradually became nonlinear at higher stress levels. Based on the bending characteristics of isochronous stress–strain curves, the ranges of the long-term strengths of rock should be determined, as shown in Fig. 12. According to this method, the ranges of the long-term strengths of the different damaged specimens are obtained and are listed in Table 6. Table 6 shows that the long-term strength of each specimen covers a small stress range. Therefore, the average values of the stress ranges listed in Table 6 can be considered to be the long-term strengths of the damaged sandstone specimens. The relationship between the average long-term strength and the instantaneous damage is shown in Fig. 13. It can be seen that the long-term strength of the rock decreases as the instantaneous damage increases under the lower confining pressure. However, the instantaneous damage has little effect on the long-term strength under the higher confining pressure.

**Table 6:** Range of long-term strength of specimens with different instantaneous damage under different confining pressure.

Confining pressure (MPa)	Range of long-term strength (MPa)			
	#D_1_	#D_2_	#D_3_	#D_4_
5	46.0–49.5	45.6–49.1	41.3–44.2	40.2–43.0
10	55.2–57.6	53.5–56.1	53.5–55.2	53.5–55.2
20	86.1–94.0	86.3–93.0	85.0–91.4	84.0–92.6

### Creep contribution factor curve

During creep tests, materials will undergo viscoelastic deformation or viscoplastic deformation under different stress conditions. It is widely recognized that only the transient creep stage will be observed when the creep stress level is below the long-term strength of the rock. Thus, the viscoelastic deformation in the transient creep stage will become stable within this duration. When the creep stress level surpasses the long-term strength, the steady-state creep stage, which produces viscoplastic deformation, will occur after the transient creep stage. In addition, the creep strain and the creep rate of the rock have been found to be correlated with the accumulation of viscoplastic deformation in multi-loading creep tests^6,16^. Therefore, the creep strain or creep rate can be used as an index to determine the long-term strength. In this study, we defined a creep contribution factor to explore the contribution of the creep to the total deformation:

3 $$k = \frac{{\varepsilon_{{\text{c}}} }}{{\varepsilon_{{\text{t}}} }} \times 100\%$$

where *k* is the creep contribution factor, ε_c_ is the creep strain, and *ε*_t_ is the total strain.

**Figure 14:**
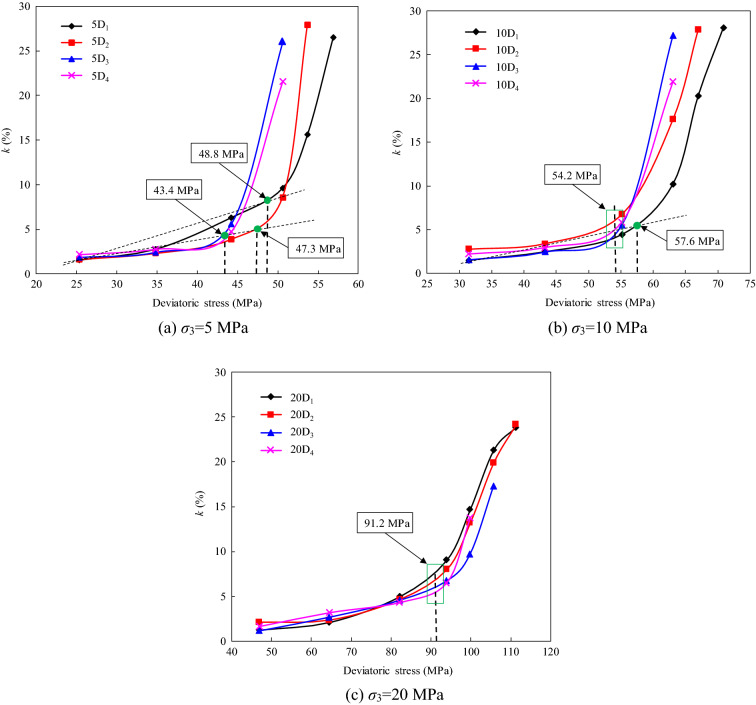
Relationship between contribution factor of creep and stress level for rock specimens with different instantaneous damage.

It can be seen from Eq. (3) that an increase in *k* indicates an increase in the time-dependent deformation. Based on the characteristics of the strain–time curves shown in Fig. 5, it can be inferred that the creep contribution factor will hardly change with increasing time at low creep stress levels, but it will change significant with the emergence of steady-state and accelerated creep. In view of this, the threshold stress of the steady-state creep stage can be determined by analyzing the variations in the creep contribution factor with creep stress at each loading level. Figure 14 shows the variation in *k* at each stress level for the specimens with different degrees of instantaneous damage under different confining pressures. The results indicate that there is an inflection point, which divides the curve into two sections, a slowly growing linear segment and a rapidly growing nonlinear segment. Accordingly, when a straight line parallel to the longitudinal axis is drawn through the inflection point, the stress corresponding to the intersection of the straight line and the transverse axis can be regarded as the long-term strength of the rock.

Using this method, the long-term strengths of the specimens with different degrees of instantaneous damage under different confining pressures were accurately identified (Table 7). The results show that the instantaneous damage of the rock has an effect on the long-term strength under low confining pressures, but this effect gradually disappears with increasing confining pressure. For example, when the confining pressure is 5 MPa, the long-term strengths of the specimens with four different degrees of instantaneous damage are 48.8, 47.3, 43.4, and 43.4 MPa, respectively. However, the long-term strengths of these damaged specimens are all 91.2 MPa under a confining pressure of 20 MPa. Moreover, it can be seen that the long-term strength obtained by analyzing the creep contribution factor curves is consistent with the range of the long-term strength determined from the isochronous stress–strain curve. This proves that the method of using the creep contribution factor curve to determine the long-term strength of the rock is reliable.

**Table 7:** Long-term strength of the damaged sandstone specimens determined by the creep contribution factor curve method.

Confining pressure (MPa)	Long-term strength (MPa)			
	#D_1_	#D_2_	#D_3_	#D_4_
5	48.8	47.3	43.4	43.4
10	57.6	54.2	54.2	54.2
20	91.2	91.2	91.2	91.2

### Discussion

In this study, the long-term strengths of specimens were determined using two different methods. According to the results, the isochronous stress–strain curve can be used to satisfactorily identify the range of the long-term strength of a rock. However, in order to accurately determine the value of the long-term strength based on the results of multi-loading creep tests, more stress levels should be tested in the tests. As can be seen from Fig. 11c,d, since the specimens experienced four creep stress levels before failure, it is difficult to identify an obvious inflection point in the isochronous curves. However, obvious inflection points can be distinguished on the creep contribution factor curves for the different stress levels, and this curve can be used to determine the long-term strength of the rock.

Based on the long-term strength values of the various damaged specimens under different confining pressures, it can be inferred that the effect of the degree of instantaneous damage of the rock on the long-term strength decreases gradually with increasing confining pressure. In contrast, at a confining pressure of 20 MPa, it seems that the initial instantaneous damage does not affect the long-term strength of the rock. Furthermore, Table 7 demonstrates that the long-term strength increases with increasing confining pressure. That is, the time-dependent behavior of the rock is significantly affected by the in situ stress environment in underground engineering.

## Macro- and micro-failure modes of pre-peak damaged sandstone

### Macroscopic failure modes

**Figure 15:**
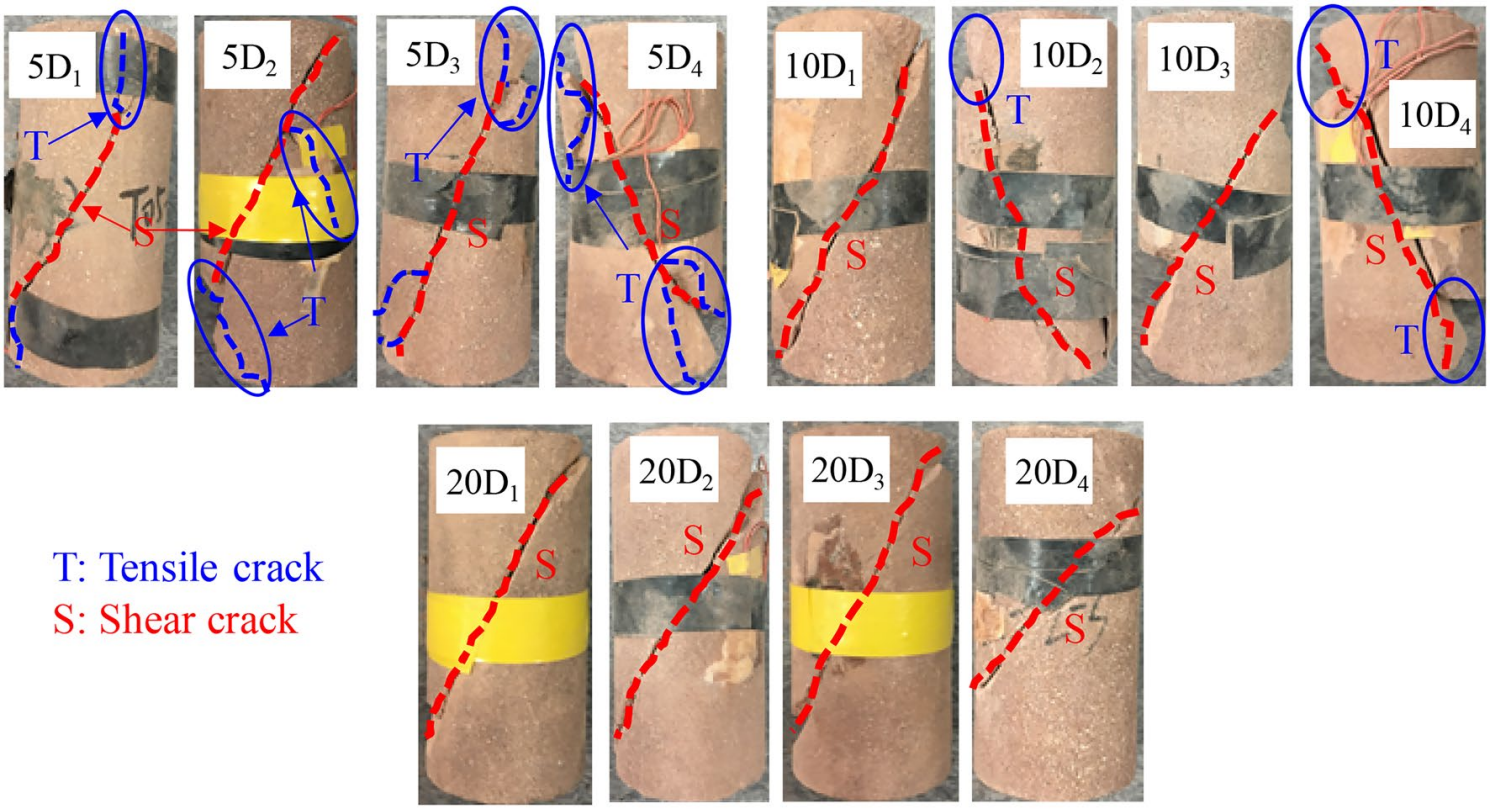
Macroscopic failure modes of damaged sandstone specimens under creep loading condition.

The macroscopic failure mode of rock under conventional triaxial compression is the result of the initiation, evolution, expansion, and penetration of the internal micro-cracks, and it contains abundant rock mechanics information. Figure 15 shows the macroscopic failure mode of the damaged sandstone specimens under multi-stage creep loading at confining pressures of 5, 10, and 20 MPa. It can be seen that the macroscale failure patterns of the sandstone specimens can be divided into two primary categories: a shear-dominated failure mode, and a mixed shear-tensile failure mode. The specimen with a mixed shear-tensile failure mode contains a main fracture, which is dominated by shear deformation, and several branch fractures. The branch fractures appear in the end of the specimen and are formed by the tensile propagation of the local microcracks. A single macroscopic shear crack crosses the specimen, and a large amount of plastic deformation occurs before failure under the shear-dominated failure mode.

It should be noted that the initial instantaneous damage of the specimen does not affect its macro-failure characteristics under the same confining pressure. However, the results show that the confining pressure is the main factor that determines the failure mode. When the confining pressure is 5 MPa, there are mainly macroscopic cracks throughout the surface of the specimen after the creep failure stage, and there are several branch cracks parallel to the axial loading direction of the specimen, indicating a typical mixed shear-tensile failure mode. When the confining pressure is 10 or 20 MPa, a fracture forms in the specimen due to the shear deformation and failure, which divides the specimen into two parts with approximately equal volumes. Therefore, it can be inferred that as the confining pressure increases, the macro-failure mode of the creep gradually changes from the mixed shear-tensile failure mode to the shear-dominated failure mode.

### Micro-fracture morphology

The microstructure of the rock specimens experienced a complex microscopic failure process from damage accumulation to fracturing under the creep load and formed a microscopic fracture surface morphology after creep failure. Numerous studies have pointed out that the macroscopic deformation and failure of a rock is essentially a nonlinear superposition of the internal microscopic deformation and the expansion and nucleation of microscopic defects^43–45^. Therefore, the microscopic fracture mechanism of a rock can be determined by studying the microscopic morphology characteristics of a specimen containing fracture surfaces.

**Figure 16:**
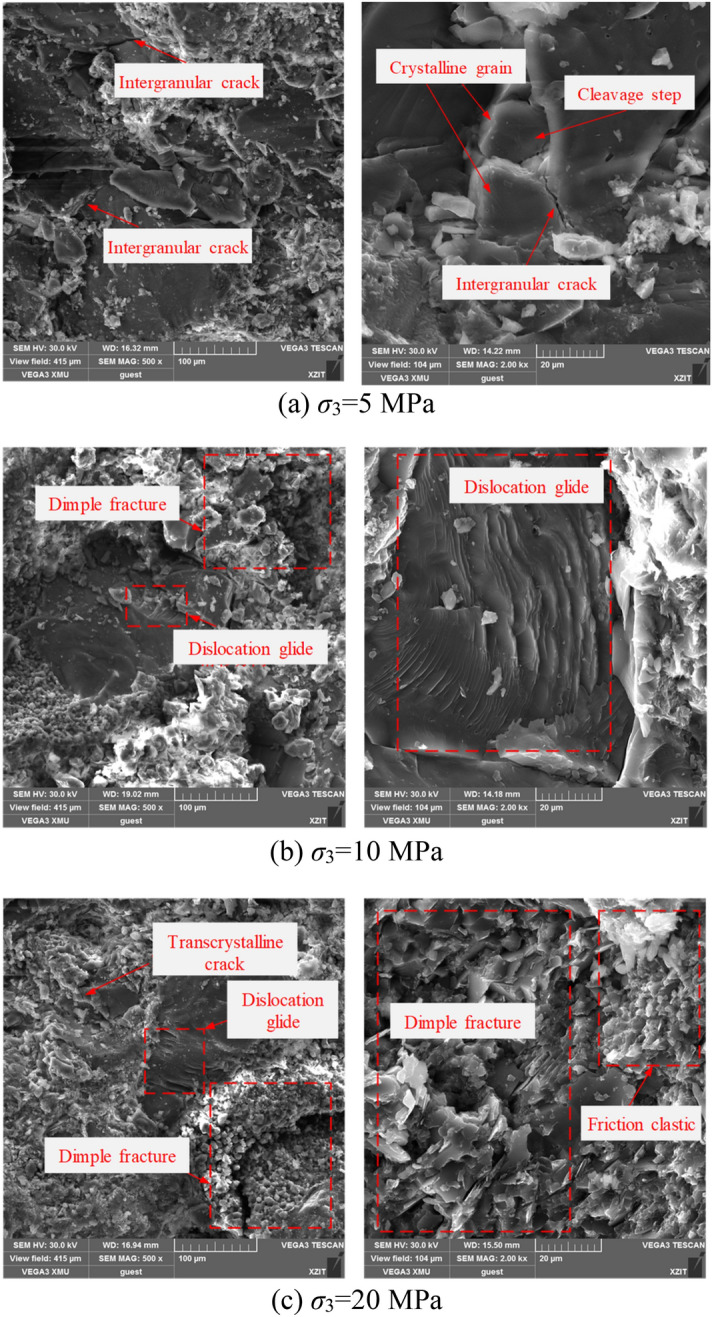
Fracture morphology of damaged sandstone under different confining pressures.

SEM is a technique for obtaining scanning images of surface features by processing the physical signals reflected from the object’s surface, and it has been widely used in microstructure analysis of solid materials from the micron-scale to the nano-scale. Many types of microstructure characteristics of fracture surfaces can be observed via SEM. Since only the local morphological features of the rock fragments can be observed at the microscopic scale, representative photos were selected from a number of micrographs of fractures under different magnifications (Fig. 16).

It was found that the fracture morphology of the sandstone after creep failure under different confining pressures is generally characterized by ductile fracture. When the confining pressure was 10 and 20 MPa, numerous dimple fracture and dislocation glide reflecting ductile fracturing were observed locally in the fracture. However, at low confining pressure (such as confining pressure of 5 MPa), the micro-morphologies of cleavage step and intergranular crack representing brittle fracture characteristics could be also observed on the fracture surfaces of the sandstone. It is evident that the micro-fracture mode of the sandstone gradually changed from a mixed brittle and ductile fracture mode to a single ductile fracture with increasing confining pressure. Combined with the macroscopic failure modes of sandstone, it can be found that the confining pressure is the key factor causing the failure mode of rock changed from brittle fracture to ductile fracture.

## Conclusions

To investigate the characteristics of creep and the long-term strength of pre-peak damaged rock under triaxial compression, a series of multi-stage creep tests on sandstone specimens with a controllable degree of pre-damage were conducted under different confining pressures. The main conclusions of this study are as follows.

1. The creep stress is a key factor affecting the time-dependent deformation of a rock under a given confining pressure. At a low-stress level, the deformation tends to become stable after the transient creep stage. The steady-state creep and the accelerating creep stages appear at high-stress levels, and the time-dependent deformation becomes unstable until the rock specimen fails. In addition, the steady-state creep rate increases exponentially with increasing creep stress.
2. The pre-peak instantaneous damage of the rock has little effect on the time-dependent deformation at low-stress levels. However, at the creep failure stress level, the instantaneous damage, and creep damage jointly affect the development of the deformation.
3. Under the same confining pressure, the creep failure times of specimens with different degrees of instantaneous damage are significantly different. This indicates that the larger the instantaneous damage is, the earlier the creep failure occurs, and the stress level of creep failure may be different. Furthermore, there is a strain threshold for the accelerating creep stage for specimens with different degrees of instantaneous damage under a given confining pressure, and the strain threshold increases with increasing confining pressure.
4. The stress corresponding to the inflection point of the creep contribution factor curve is considered to be the long-term strength. When the confining pressure is less than 10 MPa, the long-term strength decreases gently as the degree of instantaneous damage increases; however, the long-term strength is not significantly affected by the instantaneous damage under high confining pressures.
5. The macro-failure mode and the micro-fracture morphology are both affected by the confining pressure. Under low confining pressures, the main fracture is dominated by shear deformation, and the branch fractures in the end of the specimen are formed by the tensile propagation of local microcracks. Under high confining pressures, the macro-failure mode of sandstone is mainly shear failure, and its micro-fracture morphology reflects the mixed fracture characteristics of brittleness and toughness.

## Data Availability

The datasets used and analyzed during the current study available from the corresponding author on reasonable request.
